# William R. Warner & Co.’s Exhibit at Saint Louis

**Published:** 1904-07

**Authors:** 

**Affiliations:** Philadelphia


### CORRESPONDENCE.

### William R. Warner & Co.'s Exhibit at Saint Louis.

*Editor Buffalo Medical Journal:*

Sir—We have an interesting exhibit at the Saint Louis Exposition located in the Palace of Liberal Arts, and, inasmuch as no general information is given out as to where pharmaceutical and chemical displays are shown, we have thought it would be of general interest to your subscribers to have the fact laid before them, not only as to our particular exhibit, but the class generally. If it is consistent with your practices, we would like to have you give prominence in your pages to the facts presented.

Wm. R. Warner & Co.

Philadelphia, May 25, 1904.

---

Cardiac Hypertrophy.—Cactus grandiflorus *(Flint's Encyclopedia)* may be called for when the heart's action is much excited, with a sensation as if the heart were grasped or constricted. Hare advocates perfect rest; moderate amounts of nonstimulating foods —no wines or coffee; inunction of small amounts of veratrine ointment or application of belladonna plaster over pericardium if heart's action very excessive; aconite or veratrum viride internally. —*Denver Med. Times.*

